# An Exploratory Study of the Metabolite Profiling from Pesticides Exposed Workers

**DOI:** 10.3390/metabo13050596

**Published:** 2023-04-27

**Authors:** Daniela Magalhães Nolasco, Michele P. R. Mendes, Luiz Paulo de Aguiar Marciano, Luiz Filipe Costa, Adriana Nori De Macedo, Isarita Martins Sakakibara, Alessandra Cristina Pupin Silvério, Maria José N. Paiva, Leiliane C. André

**Affiliations:** 1 Department of Clinical and Toxicological Analysis, Faculty of Pharmacy, Federal University of Minas Gerais (UFMG), Belo Horizonte 31270-901, MG, Brazil; 2 Toxicants and Drugs Analysis Laboratory, Faculty of Pharmacy, Federal University of Alfenas (UNIFAL), Alfenas 37130-001, MG, Brazil; 3 Chemistry Department, Federal University of Minas Gerais (UFMG), Belo Horizonte 31270-901, MG, Brazil; 4 Faculty of Pharmacy, Professor Édson Antônio Vellano University (UNIFENAS), Alfenas 37132-440, MG, Brazil

**Keywords:** pesticides, untargeted metabolomic, UPLC-Q-TOF-MS, occupational toxicology, plasma, urine

## Abstract

Pesticides constitute a category of chemical products intended specifically for the control and mitigation of pests. With their constant increase in use, the risk to human health and the environment has increased proportionally due to occupational and environmental exposure to these compounds. The use of these chemicals is associated with several toxic effects related to acute and chronic toxicity, such as infertility, hormonal disorders and cancer. The present work aimed to study the metabolic profile of individuals occupationally exposed to pesticides, using a metabolomics tool to identify potential new biomarkers. Metabolomics analysis was carried out on plasma and urine samples from individuals exposed and non-exposed occupationally, using liquid chromatography coupled with mass spectrometry (UPLC-MS). Non-targeted metabolomics analysis, using principal component analysis (PCA), partial least squares discriminant analysis (PLS-DA) or partial least squares discriminant orthogonal analysis (OPLS-DA), demonstrated good separation of the samples and identified 21 discriminating metabolites in plasma and 17 in urine. The analysis of the ROC curve indicated the compounds with the greatest potential for biomarkers. Comprehensive analysis of the metabolic pathways influenced by exposure to pesticides revealed alterations, mainly in lipid and amino acid metabolism. This study indicates that the use of metabolomics provides important information about complex biological responses.

## 1. Introduction

Pesticides constitute a heterogeneous category of chemicals designed specifically for the control of pests, weeds or plant diseases. The human population is environmentally and occupationally exposed to various pesticides, whose applications have increased significantly in recent years. Despite being necessary for pest control, they represent a serious problem for human health and the environment, mainly due to the lack of control and inspection of exposure. Several health and environmental risks are associated with these chemicals, which can lead to acute or chronic toxic effects, including infertility, hormonal disorders, psychiatric disorders and cancer. For these reasons, pesticides have been the object of studies, both for the damage they cause to human health as well as for the damage to the environment and the emergence of resistance in organisms [1,2,3,4,5,6].

Considering the potential risk to human health, biomonitoring is a useful tool to assess the risk of workers exposed to pesticides. Usually, the determination of the activity of cholinesterase enzymes is used for this purpose, but in many cases, this determination has been shown to be insufficient to characterize the harmful effects resulting from exposure to different pesticides or mixtures of them. In addition, this biomarker is ineffective for the early indication of deleterious effects induced by toxic agents [7,8,9,10]. In this sense, characterizing the metabolic response to pesticides and how they influence human health becomes a very important step, and metabolomics presents itself as a useful alternative capable of achieving this goal.

Metabolomics has been used in several areas to understand cell functioning and biological changes in organisms. This technique provides an integrated view of biochemistry in complex systems and, in the area of occupational and environmental toxicology, is especially useful in the identification of new biomarkers of chemical contaminants, in addition to exhibiting great potential for clarifying the mechanisms of toxicity of these agents as well as investigating metabolism and other possible biological interactions. There are some challenges involved with metabolomics, such as the analytical process, which is critical, in addition to difficulties involved with reproducing the data necessary for the replication of studies or even their continuity after the discovery of biomarkers [11,12,13,14].

Based on metabolite coverage, there are different analytical approaches to metabolomics, classified as targeted analysis and untargeted analysis. Non-targeted metabolomics has been successfully applied in environmental and occupational toxicology studies, providing relevant information on the mechanisms of toxic action and even identifying biomarkers [15,16,17,18,19,20].

Due to the complexity of biological systems and the physicochemical diversity of all metabolites, it is necessary to use different analytical techniques; the main ones are nuclear magnetic resonance—NMR—and mass spectrometry—MS—coupled with different techniques of separation, such as liquid chromatography, gas chromatography or capillary electrophoresis. Liquid chromatography is currently recognized as the most used technique in global metabolomics [11,21,22,23]. However, there are few studies on the elucidation of the metabolic profile in individuals exposed to pesticides.

Thus, the objective of the present study was to carry out an exploratory experimental analysis to identify whether there are differences in the plasmatic and urinary metabolic profile of individuals exposed and not exposed occupationally to pesticides, using ultra-performance liquid chromatography coupled with an ionization mass spectrometer by quadrupole electrospray with time of flight (UHPLC-ESI-Q-TOF-MS). Multivariate statistical analysis was used to evaluate the data and identify discriminating metabolites between the two groups, with the purpose of providing important information about complex biological responses and discovering possible candidates for biomarkers that can be used in clinical practice to promote biomonitoring.

## 2. Materials and Methods

### 2.1. Population and Sample Collection

The study population consisted of 40 male individuals, aged between 20 and 62 years, who were divided into two groups. The exposed group consisted of 20 rural workers from the city of Paraguaçu, Minas Gerais, Brazil, who were occupationally exposed to pesticides. The control group consisted of 20 male volunteers, living in Belo Horizonte and region, Minas Gerais, Brazil, with no history of occupational exposure to xenobiotics.

All volunteers who participated in this study signed a free and informed consent form before collecting the biological material. Subsequently, 10 mL of peripheral blood was collected by venipuncture and 50 mL of urine was collected in sterile bottles, in an average period of 7 days after pesticide application. All samples were immediately transported, in a refrigerated box, to the laboratory, where they were stored at −70 °C until analysis.

### 2.2. Ethical Aspects of Research

This study was submitted and approved by the ethics and research committee of the Federal University of Minas Gerais—UFMG (CAAE: 39339720.0.0000.5149, opinion number: 5.473.586).

### 2.3. Standards and Reagents

The chromatographic-grade solvents methanol, formic acid, 2-propanol, acetonitrile and sodium formate were obtained from Sigma-Aldrich^®^ (San Luis, MO, USA).

### 2.4. Sample Preparation

#### 2.4.1. Plasma Preparation

Plasma samples were frozen at −70 °C to promote metabolic quenching. To perform the analysis, the samples were thawed and vortexed; then a 100 µL aliquot of plasma was extracted, using 300 µL of a chloroform: methanol (2:1, *v*/*v*) mixture. The samples were vortexed for 30 s and centrifuged at 14,000× *g* for 10 min at 4 °C, and then 5 µL of the supernatant was injected into the UPLC-QTOF-MS. Quality-control samples (QCs) were prepared by mixing 20 µL of each plasma sample and processed in the same way. Sample blanks were also prepared, containing all reagents used in the preparation except for plasma [24,25].

#### 2.4.2. Urine Preparation

Urine samples were thawed and vortexed for 30 s. Then, 100 µL was pipetted into 125 µL of methanol in Eppendorf microtubes and vortexed again for 30 s. Samples were centrifuged at 14,000× *g* for 10 min at 4 °C for protein precipitation. In sequence, 200 µL of the supernatant was transferred to properly labeled flasks and 300 µL of an aqueous solution containing 0.1% methanoic acid was added. Quality-control samples (QCs) were prepared by mixing 20 µL of each urine sample and processed in the same way. Sample blank preparations were also performed, where all reagents except plasma were pipetted [25].

### 2.5. Analytical System

After preparation, plasma and urine samples were analyzed by ultra-performance liquid chromatography (Shimadzu^®^, Tokyo, Japan) coupled with a mass spectrometer (Bruker^®^, Billerica, MA, USA). For chromatographic separation, a C_18_ reversed-phase column (100 mm × 2.1 mm 1.8 µm particle) was used, and temperature was maintained at 40 °C. A linear gradient elution of solvent consisting of (A) water with 0.1% formic acid and (B) acetonitrile with 0.1% formic acid was performed as follows: 98–2% A (0–8 min), 9010% A (8–14 min), 50–50% A (14–14.50 min), 0–100% A (14.50–16.50 min) and 98–2% A (16.50–20 min) at a flow rate of 0.4 mL/min. More details are described in Table S1. The injection volume was 5 µL for all samples, and they were analyzed under the same analytical conditions, randomly, to avoid uncertainties and artefacts related to the injection order and to prevent the effect of gradual changes in instrument sensitivity over entire batches.

The mass spectrometer was operated in positive mode, using electrospray ionization (ESI) and collision energies of 20 and 50 eV (each 50% of the time). The source conditions were set to nebulizer gas (N_2_) at 5.0 bar, drying gas (N_2_) at 9.0 L/min, dry gas temperature at 200 °C, capillary voltage at 4.5 KV; the ionization source was set to 4500 V. A solution of hexakis (2,2-difluoroethoxy) phosphazene in 2-propanol was used for lock mass calibration, and a sodium formate solution was used for calibration before analysis. Ions in the range of 20 to 1000 m/z were monitored, with an acquisition rate of 4 Hz. In order to monitor the stability and reproducibility of the analytical conditions, 3 blank solvents and 5 QC samples were injected at the beginning of the sample test, another 3 blank solvents were injected at the end of the test and 1 QC sample was injected every 5 study samples. Data were acquired using Hystar Application^®^ version 3.2 and OtofControl^®^ software version 3.4 (Bruker Daltonics Corporation^®^, Billerica, MA, USA).

### 2.6. Detection and Identification of Non-Target Metabolites

Data files obtained by UPLC-MS were converted to mzML format using the Proteowizard^®^ software (http://proteowizard.sourceforge.net/download.html (accessed on 24 September 2022)). The statistical software R^®^ version 4.2.1 and the XCMS Bioconductor^®^ package version 3.12 (http://bioconductor.org/packages/release/bioc/html/xcms.html (accessed on 24 September 2022)) were used for pre-treatment of the data, including baseline correction, denoising, deconvolution, peak alignment and generating the data matrix. The analysis of the results was performed using the centWave algorithm in XCMS^®^, with snthresh = 5.0, mzwid = 0.01 and bw = 5. Urine-related data were normalized by the corresponding urinary creatinine concentration, and plasma sample results were normalized by the median of intensities. Data filtering was applied to all data to remove features with relative standard deviation (RSD) higher than 30% from the subsequent analysis. After that, logarithmic transformation was used as well as scaling by mean centering. This processing was performed using the MetaboAnalyst^®^ software version 5.0 (https://www.metaboanalyst.ca/ (accessed on 24 September 2022)).

The putative identification of the metabolites recognized by UPLC-QTOF-MS was performed by searching for m/z’s in public databases, such as the Human Metabolome Database (HMDB^®^) and Metlin^®^, in addition to the CEU Mass Mediator platform—where a search is carried out in different databases simultaneously (Kegg, HMDB^®^, LipidMaps^®^, Metlin^®^)—considering a maximum mass error of 5 ppm and the adducts [M+H]^+^, [M+NH_4_]^+^ and [M+Na]^+^.

### 2.7. Statistical Analysis

The plasma and urine results obtained were subjected to multivariate statistical analysis using unsupervised chemometric methods, such as principal component analysis (PCA), and supervised methods, such as partial least squares discriminant analysis (PLS-DA) and orthogonal discriminant analysis by partial least squares (OPLS-DA). Cross-validation was used to validate supervised models. All these analyses were performed in MetaboAnalyst^®^ 5.0. From the PLS-DA and OPLS-DA models, discriminating molecular features between the groups were identified using the variable importance in projection (VIP) and volcano plot classification, adopting the criteria of VIP > 1.5; FDR < 0.01, *p*-values between analyzed groups < 0.05 and fold change > 2.0.

Descriptive statistics were also applied to evaluate numerical variables, such as mean, standard deviation and median. The Shapiro–Wilk and Anderson–Darling tests were used to verify whether the variables had a Gaussian normal distribution. Categorical variables were evaluated in terms of frequency and percentage. To verify the hypotheses of association between variables with normal distribution, the t-test was used; for the others, the Mann–Whitney test and Fisher’s exact test were used. An association was considered statistically significant when the *p*-value was less than 0.05. The software used in the analyses was Graphpad Prism^®^ version 9.0.0 for Windows (GraphPad Software, San Diego, CA, USA, 2020).

## 3. Results and Discussion

### 3.1. Population Characteristics

The main characteristics and distribution in relation to the study population are presented in Table 1. The mean age of individuals in the control group was 39 years, ranging from 20 to 62 years, and the mean age of the exposed group was 45 years, ranging from 22 to 62 years. Most volunteers in this group (70%) worked with family agriculture on small and medium-sized properties, while 75% of the control group had an administrative function. All participants in the exposed group had been in direct contact with pesticides for more than three years, with 44% of them reporting more than 20 years of exposure.

Comparisons made between the exposed and control groups for the variables schooling (time of study), age group and occupation showed that only for the variable occupation was there a significant association (*p* < 0.001), indicating that there is a higher prevalence of individuals with administrative function among individuals in the control group, while among those exposed, the highest prevalence was for family agriculture and applicator.

### 3.2. Identification of Plasma Metabolic Profile of Individuals Occupationally Exposed to Pesticides

**Figure 1:**
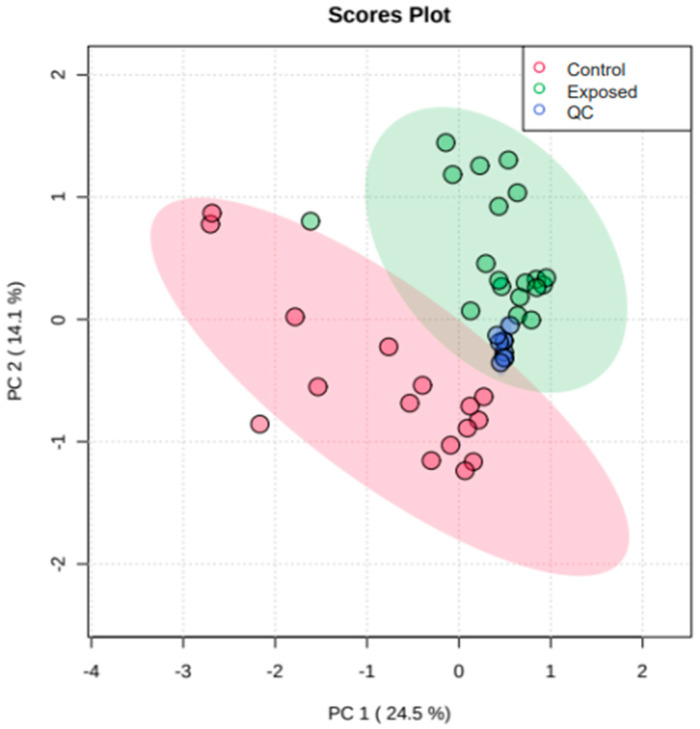
Score graph of principal component analysis (PCA) models for plasma samples from individuals exposed and non-exposed occupationally to pesticides as well as quality control samples (QCs).

After the processing steps, multivariate statistical methods were applied to the data matrix. These were initially evaluated using the unsupervised pattern recognition method principal component analysis (PCA) with quality control (QC) prediction, to find similarities and differences between the samples and verify the presence of trends or outliers. Figure 1 shows a good separation of samples from individuals occupationally and non-occupationally exposed to pesticides, reflecting differences in their metabolic profiles. PCA analysis was used to assess the quality of data acquisition, and the strong clustering of QC samples, observed in the center of the graph (Figure 1), indicated analytical stability, quality and reliability of the data obtained.

**Figure 2:**
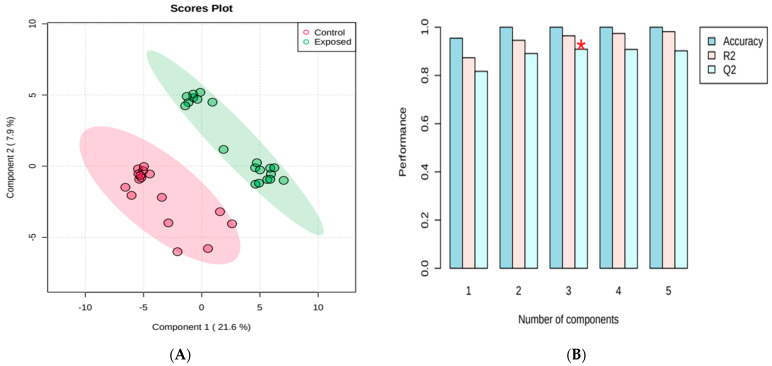
Partial least squares discriminant analysis (PLS-DA) indicating effective discrimination between the group of occupationally exposed individuals and the control group (volunteers not occupationally exposed to pesticides) (**A**) and PLS-DA classification by cross-validation, using different number of components. The red star indicates the best classifier (**B**).

The results were then evaluated by the supervised pattern recognition method discriminant partial least squares analysis (PLS-DA) to model the differences between the metabolites responsible for the separation of groups. The score graph (Figure 2) shows an effective separation between the plasma samples from the control group versus the exposed group. In order to test the validity of the constructed model, a cross-validation test was carried out, which can be seen in Figure 2B. The cross-validation results were R^2^ = 0.98, Q^2^ = 0.92, which shows an adequate fit of the data to the model, and therefore it is considered a highly accurate model. Usual values for biological experiments are Q^2^ > 0.4 and R^2^ > 0.7 [26,27].

To maximize the separation between groups and find discriminating metabolites, the supervised partial orthogonal least squares discriminant analysis (OPLS-DA) classification method was also used. The score graph obtained is shown in Figure S1.

**Figure 3:**
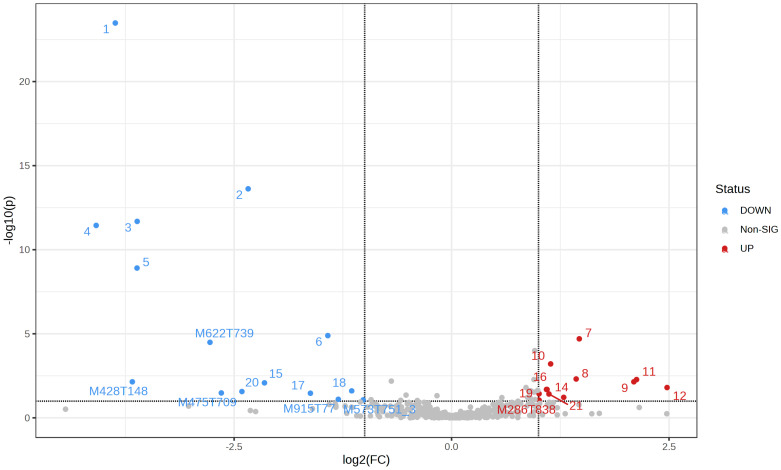
Volcano plot applied to data from plasma samples of individuals exposed and not exposed occupationally to pesticides. Note: each point on the plot represents a molecular feature. The red dots indicate an increase in features in the control group and the blue dots, a decrease in the same group. The gray dots represent a lack of distinction between the groups. The dots identified by numbers correspond to the compounds listed in Table 2.

Using volcano plot analysis, which allows selection of significant features based on biological significance and statistical significance, differential metabolites were identified, based on criteria of fold change > 2.0, FDR < 0.01, VIP > 1.5 and *p*-value < 0.05. The VIP value, obtained in the OPLS-DA model, indicates the importance of each metabolite in the discrimination between groups. Thus, using all these criteria, 14 molecular features were found with lower and 11 with higher results when comparing the control group with the exposed group, which is represented in Figure 3.

Based on the results obtained, 25 molecular features that contributed most to the discrimination between groups were selected. The identification of four of these features was not possible due to non-recognition of the respective m/z by the databases used. The results of this evaluation and the possible identification are compiled in Table 2, where the VIP score, *p*-value, fold change and chemical classification of the compounds are also presented.

To evaluate the performance of these compounds as potential biomarkers in the evaluation of toxicity induced by pesticides, the ROC curve (receiver operating characteristic) was used, which is a graphic representation that illustrates the performance of a compound related to its discriminatory power. An area under the curve (AUC) of 0.5 indicates that a substance has no discriminating ability, while an AUC of 1.0 represents a test with perfect discrimination. AUC values > 0.85 are considered acceptable for clinical applications [28]. Based on these results, 16 candidates performed satisfactorily, including 1-[2-chloro-4-(4-chlorophenoxy)phenyl]-2-(1,2,4-triazol-1-yl)ethanol (AUC = 1.0), PG(18:3(9Z,12Z,15Z)/22:4(7Z,10Z,13Z,16Z)), with AUC = 0.998; Ceramide (AUC = 0.99) and Sphingomyelin (AUC = 0.90). On the other hand, five compounds, 5-O-b-D-glucopyranoside, 13,14-Dihydro PGE1, Val-Gly-Asp, phosphatidylethanolamine and phosphatidylcholine, had AUC < 0.85. The ROC curves of some possible biomarkers are shown in Figure S2.

According to the ROC curve results, the most discriminating compound between the groups was 1-[2-chloro-4-(4-chlorophenoxy)phenyl]-2-(1,2,4-triazol-1-yl)ethanol (AUC = 1), a metabolite of difenoconazole, a fungicide of triazole class widely used by the workers involved in this study, according to their reports.

According to the literature, triazoles act not only on the target enzymes of the CYP51 family (lanosterol-14α-demethylase), necessary for the biosynthesis of ergosterol, but also on other enzymes of the cytochrome P450 family. Exposure to triazole fungicides can trigger endocrine disruption due to inhibition of aromatase activity, oxidative stress, cell apopthesis and inflammatory reactions. A product of CYP51 demethylation in humans is cholesterol, which is required for the synthesis of bile acids, mineralocorticoids, glucocorticoids and sex steroids [19,29].

Jiang et al. [30] and Teng et al. [29] used transcriptomics and metabolomics by LC-MS/MS to verify the toxicity of difenoconazole in zebrafish. The analysis of metabolic pathways, after exposure to this pesticide, detected changes in amino acid metabolism, including glutamate, lipid metabolism, energy metabolism and nucleotide metabolism, among others. Both authors stated that fungicides disrupted lipid metabolism at biochemical, transcriptomic and metabolomic levels and concluded that the two techniques, when used together, may be useful to verify the toxicological effects of difenoconazole in zebrafish and to assess the risks of xenobiotics in aquatic organisms.

An interesting evaluation was performed by Van Meter et al. [31] on the influence of exposure to pesticide mixtures on the metabolomic profile of frogs. They tested three herbicides, an insecticide and a triazole fungicide, exposing the amphibians to a single compound and mixtures of these. They observed that the metabolites and pathways impacted were different between treatments, indicating that the modes of action of xenobiotics may change depending on chemical interactions with other toxicants. However, the authors reported that regardless of the mode of action, some metabolites commonly altered by these mixtures were amino acids and lipids, which are critical for protein synthesis, DNA structure and replication and as a response to oxidative stress. Similar results were observed in our study.

### 3.3. Analysis of Metabolic Pathways Affected by Exposure to Pesticides Based on Plasma Metabolomics Results by UPLC-QTOF-MS

**Figure 4:**
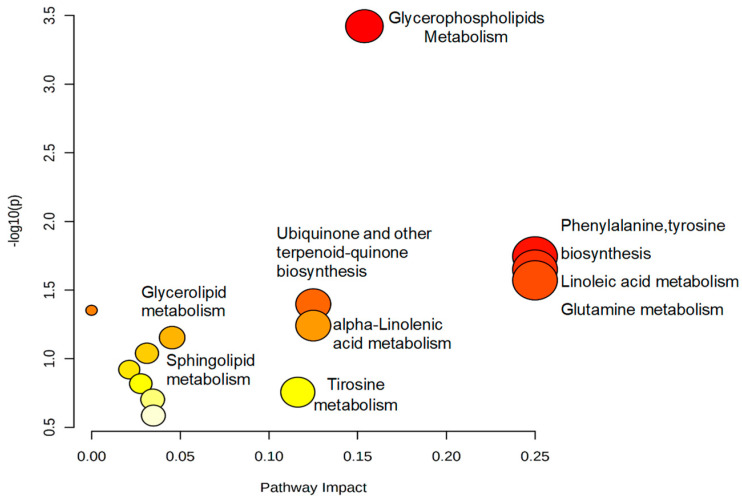
Representation of the metabolic pathways involved in the biological response associated with exposure to pesticides. The colors, varying from yellow to red, represent metabolites with different levels of significance.

After identifying the discriminating metabolites, we sought to understand the metabolic pathways involved in the biological response generated by exposure to pesticides. For this, the selected variables were subjected to metabolic pathway analysis, which was conducted using the MetaboAnalyst^®^ software (https://www.metaboanalyst.ca/ (accessed on 24 September 2022). The results are represented in Figure 4, which shows all corresponding pathways according to pathway impact values and *p*-values from the pathway enrichment analysis. The size of the circle represents the influencing factor, and the color identifies the importance of the pathway for understanding the biological response. Therefore, the larger and red circles are considered the most important pathways, i.e., the most disturbed ones.

The alterations were found in fourteen metabolic pathways, as shown in Figure 4; the main ones were glycerophospholipid metabolism, phenylalanine/tyrosine biosynthesis, linoleic acid metabolism and glutamine metabolism.

#### 3.3.1. Metabolism of Glycerophospholipids and Linoleic Acid

Lipids are organic compounds that can be divided into eight categories, according to the classification proposed by LIPIDMAPS (http://www.lipidmaps.org (accessed on 26 November 2022)): fatty acids, glycerolipids, glycerophospholipids, sphingolipids, sterol lipids, prenollipids, saccharolipids and polyketides. Studies in the literature report that this chemical group plays an important role in living systems due to its biological functions and effects on human health, such as energy storage, cell membrane structure, cell communication, regulation of biological processes and relationship with cardiovascular and other chronic diseases [14,18].

In this study, alterations in the classes of glycerolipids, sphingolipids and fatty acids were identified and corroborated by studies found in the literature.

Wang et al. [32] and Weng et al. [33] studied the toxicity of epoxiconazole triazole exposure in zebrafish, and both reported that triazoles can induce an imbalance of oxidative homeostasis, causing disturbances in energy metabolism, lipid metabolism and amino acid metabolism. Yang and colleagues [34] explored the association between maternal exposure to 37 pesticides from different classes and gestation length in rats. By metabolomic analysis, the authors realized that the most affected pathway was glycerolipid metabolism and concluded that these positive changes were related to exposure to pesticides.

Nguyen et al. [14] evaluated the neurotoxicity of the fungicides strobilurin, azoxystrobin and trifloxystrobin in human neuroblastoma cells. They stated that the concentration of some lipids was increased when cells were exposed to these toxicants when compared to the control group, such as phosphatidylcholine, phosphatidylethanolamine, triglycerides, PI (14:1_16:0), ceramides and carnitines, among others. The authors said that these compounds are essential components in neuronal and mitochondrial cells, and the altered expression of these lipids may be related to mitochondrial dysfunction.

Similar results were revealed by the metabolomics of this study, such as increased levels of phosphatidylcholine, phosphatidylglycerol and phosphatidylethanolamine.

The structural diversity of these glycerophospholipids plays a key role in membrane fluidity and stability. Studies report that due to the increased demand for membrane constituents, there is an increase in phosphatidylcholine synthesis in cancer cells and solid tumors. Its reduction has been observed in pathological conditions in the liver in humans, including liver failure. Regarding phosphatidylglycerol, high concentrations of this substance were detected in acute coronary syndrome and may be linked to the pathogenesis of cardiovascular diseases. As for phosphatidylethanolamine, research claims that these compounds may be related to vascular diseases and increased incidence of cancer, in addition to playing an important role in other diseases [35,36,37].

Wang et al. [38] performed untargeted integrated lipidomics and metabolomics analyses to verify the effects of imidacloprid and acetamiprid pesticides on neuronal cells. Pathway analysis demonstrated that glycerophospholipid and sphingolipid metabolism were the most affected. According to the authors, changes in the concentration of glycerophospholipids indicate changes in the composition and permeability of the cell membrane and that the increase in phosphatidylcholine found may have occurred due to disruption of the composition of the cytoskeleton and glycerophospholipids caused by the entry of xenobiotics into cells, resulting in alterations in the composition and content of phosphatidylcholines. On the other hand, exposure to imidacloprid suppressed fatty acid synthesis.

Sanchez et al. [19] studied the neurotoxicity of triazoles, propiconazole and tebuconazole in mitochondrial dysfunction and alteration of lipid metabolism in human cells. According to the authors, neurotoxicity can occur through several mechanisms, such as oxidative stress and free radical formation, mitochondrial impairment and apoptosis. In the study conducted by these authors, it was proven that triazoles act on these mechanisms and, as a biological response, lipid alterations occur. They found altered levels of phosphatidylcholine, phosphatidylglycerol and phosphatidylinositol, in addition to identifying new lipids that may be significant in neurodegenerative diseases.

Another altered metabolic pathway identified in this study was the metabolism of 9-12-octadecadienoic acid (linoleic acid) and 9-12-15-octadecatrienoic acid (α-linolenic acid). These lipids, called polyunsaturated fatty acids, play an important role in maintaining the membrane, signal transduction and anti-inflammatory properties, in addition to participating in the synthesis of hemoglobin and cell division. They are responsible for modulating transmission in the cholinergic, serotonergic and dopaminergic systems and are involved in neurotrophic support and oxidative stress by modulating the expression of genes responsible for these actions [39,40].

The most important metabolite of linoleic acid in animal tissues is arachidonic acid, and several families of eicosanoids are derived from this acid, including prostaglandins, thromboxanes and leukotrienes. In this study, plasma metabolomics showed an increase in prostaglandin E1 (PGE1) in occupationally exposed individuals.

Prostaglandins are prostanoids synthesized from arachidonic acid by cyclooxygenase enzymes (COX-1 and COX-2). They have essential homeostatic functions in renal physiology, are potent vasodilators and platelet aggregation inhibitors but are also implicated in many pathological conditions, such as inflammation, cardiovascular disease and the initiation of carcinogenesis, representing the link between inflammation and cancer. Under normal conditions, levels of prostanoids in cells are low, but during a disturbance, their concentration can be altered [13,35].

In a study carried out by Yan et al. [41], the serum metabolic profile of individuals exposed to pesticides was evaluated by LC-MS. Among the results described by the authors, changes were observed in pathways related to inflammation, including metabolism of arachidonic acid and prostaglandins, which were associated with exposure to xenobiotics. According to the authors, oxidative stress can increase the production of arachidonic acid, an inflammatory intermediate that can be converted into prostaglandins. Pabst et al. [42] performed lipidomics in patients with acute myeloid leukemia and reported changes in a large number of plasma lipids, including prostanoids. The authors showed that arachidonic acid precursors and their metabolites are positively related to disease severity and prognosis.

Tyurina et al. [43] performed an LC/MS analysis of cardiolipins in plasma from insecticide-exposed rats. Among the results found by the authors, increased levels of arachidonic acid and linoleic acid were detected and correlated with neuroinflammation and oxidative stress, causing neurodegeneration in the animals. They cited that these findings may contribute to a better understanding of the pathogenesis of Parkinson’s disease and lead to the development of new biomarkers of mitochondrial dysfunction.

Another study was developed by Birch and collaborators [44], who investigated the effects of endocrine disruption of pesticides on calcium influx in human spermatozoa. They found that these toxicants can interfere with human sperm function through effects on the Ca^2+^ channel induced, for example, by increased PGE1, causing deficiencies in capacitation, sperm motility and chemotaxis towards the ovum. According to the authors, the findings were able to relate the effects of exposure to pesticides with human fertility.

The metabolism of sphingolipids was another pathway highlighted as important in exposure to pesticides in this study. Sphingomyelin showed a lower concentration in the workers’ plasma compared to the control group, whereas ceramide was present at more concentrated levels.

Sphingolipids are physiologically related to the regulation of cell growth, differentiation, and apoptosis. Among the types of sphingolipids, ceramides are structural components of the membrane and secondary messengers in cell signaling [19,45].

Results similar to ours were found by Weng et al. [33] when studying the toxicity of exposure to triazole epoxiconazole in zebrafish. The authors believe that the activation of enzymes responsible for the conversion of sphingomyelin into ceramide (sphingomyelinases), caused by exposure, explained the decrease in sphingolipid.

Pabst et al. [42] and Robinson et al. [46] studied the relationship between reactive oxygen species (ROS) and the metabolome in acute myeloid leukemia cells. The authors detected lower levels of sphingolipids in the plasma of these individuals and, after an overall analysis of the findings, concluded that ROS are important in regulating the synthesis and/or degradation of sphingolipids.

As evidenced in the literature, the lipid group is involved in several biological processes, such as oxidative stress, inflammation, obesity and endocrine disruption. Many xenobiotics exert their toxic effects on these biological processes and, therefore, the investigation of changes in lipid metabolism becomes an important strategy to elucidate mechanisms of toxic action, in addition to the possibility of identifying new biomarkers of exposure to these chemical substances [45,47,48].

#### 3.3.2. Phenylalanine/Tyrosine Biosynthesis and Glutamine Metabolism

Amino acids are the building blocks of our body in the form of proteins and protein complexes, and many important metabolites, such as neurotransmitters, purines and pyrimidines, among others, are products of cellular amino acid metabolism [49].

In this study, some amino acids were altered in the exposed group compared to the control group. For example, tyrosine had reduced levels, while glutamate had increased levels.

The change in relation to tyrosine is important, as it is responsible for numerous functions in the body, being a precursor of several neurotransmitters such as dopamine, noradrenaline, adrenaline and thyroid hormones. This amino acid is synthesized from the hydroxylation of phenylalanine by the enzyme phenylalanine hydroxylase. According to studies reported in the literature, deficient tyrosine biosynthesis can lead to the accumulation of phenylalanine in body fluids and also to a reduction in the production of catecholamines (adrenaline, noradrenaline and dopamine). These concentration changes may be related to the occurrence of neurodegenerative and neuropsychological symptoms, such as changes in motor activity, attention deficit and hyperactivity [49,50]. Thus, its decrease could promote these effects in the population exposed to pesticides.

Gao et al. [49] studied the physiological and biochemical changes caused in larvae exposed to the insecticide Spinetoram by transcriptomic and metabolomic analyses. Among the alterations found in the metabolism of amino acids, the authors cited a decrease in tyrosine in the studied group, which consequently generated a decrease in dopamine, causing Parkinson’s disease in the exposed insects. Rodrigues et al. [51] also detected tyrosine levels reduced by exposure to pesticides in neuronal cells and correlated changes in amino acid levels with neurodegenerative conditions in patients with Alzheimer’s disease.

Ch et al. [52] analyzed the metabolic profile of the saliva and urine of male farmers exposed to pesticides. They found results similar to our study, such as changes in amino acid levels, and stated that oxidative stress due to exposure to xenobiotics caused disturbances in amino acid and energy metabolism.

Yan et al. [41] verified the hypothesis that exposure to neonicotinoid insecticides causes disorders of amino acid metabolism, lipid accumulation and oxidative stress in mice. By evaluating non-target metabolomics, they observed significant increases in some amino acids, such as phenylalanine, suggesting that tyrosine biosynthesis was disturbed. Furthermore, they claimed that the increase in branched-chain amino acids and phenylalanine also induced lipid accumulation, which was consistent with the increase in lipid compounds identified by them in animals exposed to neonicotinoids. This exposure also increased levels of glutamate, which is associated with increased energy needs. These results are in line with those found in this study.

Regarding glutamine, this amino acid is involved in the synthesis of nucleic acids, nucleotides and proteins, among others. Its metabolism involves two enzymes: glutamine synthase, which is related to the synthesis of glutamine, and glutaminase, which acts in its conversion into glutamate, the latter being an important amino acid in cell metabolism that can be converted into gamma-aminobutyric acid (GABA), glucose, urea, synthesis of other amino acids or glutathione. Studies claim that negatively regulated glutamate uptake can contribute to the accumulation of glutamate in the synaptic cleft, causing a neurodegenerative process called excitotoxicity [51,53].

Changes induced by exposure to pesticides in the brain metabolome were studied by Rodrigues et al. [51]. In this review, they reported that deviations were observed in the level of metabolites related to several metabolic pathways, including energy metabolism, mitochondrial dysfunction, lipid and amino acid metabolism. There was an increase in glutamine and glutamate levels in the brain, affecting the pathway that is considered the main regulator of glutamate levels. Wang et al. [32] and Bonvallot et al. [54] also found similar results in zebrafish exposed to epoxiconazole and in a group of pregnant rats exposed to a mixture of pesticides, respectively.

Cattani et al. [55] investigated the effects of glyphosate exposure on some neurochemical and behavioral parameters in rats. Their results showed that exposure to this pesticide caused oxidative stress, in addition to affecting cholinergic and glutaminergic neurotransmission in the hippocampus of the animals. The authors concluded that neurotoxicity induced by glyphosate after exposure involves the phenomenon of glutamate excitotoxicity, due to increased release of glutamate in the synaptic cleft, lower uptake of this by interaction of glyphosate with receptors, leading to increased ionic flow of Ca^2+^ to the hippocampus cells. These events culminate in oxidative stress, astrocytic dysfunction and depressive behavior.

#### 3.3.3. Ubiquinone Biosynthesis

Ubiquinone, or Coenzyme Q, is a ubiquitous lipid that is involved in electron transport and oxidative phosphorylation. The biosynthesis of this compound is a multienzymatic process involving several precursors and, in mammals, the group attached to ubiquinone is derived from the essential amino acid phenylalanine, which is converted to tyrosine and, later, 4-hydroxybenzoate [6,56,57]. In this study, the metabolic alteration of this pathway was evidenced by the decrease in tyrosine in the workers’ plasma.

Payet et al. [56] evaluated the initial steps in the ubiquinone biosynthesis pathway in yeast. The authors combined techniques such as isotopic labeling, chemical analogue supplementation and genetics to identify enzymes associated with the tyrosine metabolic pathway and stated that the increase in tyrosine in cell culture raised the level of ubiquinone, concluding that this amino acid is used as a precursor of Coenzyme Q and that their concentrations are directly proportional.

A study developed by Vujić et al. [57] verified toxicity signature pathways in human brain endothelial cells exposed to the herbicide Paraquat. The analysis of the metabolic pathways highlighted the metabolism of ubiquinone as the most significant pathway. According to the authors, the results suggest that the studied herbicide modulates mechanisms such as oxidative stress and pathways related to hypoxia in endothelial cells. They also mentioned that Paraquat is an inducer of oxidative stress that inhibits the complexes of the respiratory chain in the mitochondria, especially complex I, which is directly related to the metabolism of ubiquinone.

Park et al. [58] studied the intracellular metabolomic alteration of the insecticide carbofuran, using non-targeted metabolomics by LC-MS. They observed alterations in the metabolism of amino acids, nucleosides (purine and pyrimidine) and ubiquinone biosynthesis. The metabolic pathways related to 4-hydroxybenzoate production and ubiquinone biosynthesis showed significant differences between the two groups studied, and the authors concluded that these changes were probably related to the response to oxidative stress induced by carbofuran. Considering the results of the study, the authors highlighted the importance of non-targeted metabolomics as a good strategy to identify intracellular alterations after exposure to xenobiotics.

The metabolomic analysis of the plasma of this study population demonstrated that several metabolites were significantly modulated by exposure to pesticides. The observed metabolic changes are consistent with studies found in the literature and may be associated with impaired membrane function, oxidative stress, inflammation, mitochondrial dysfunction and endocrine disruption.

### 3.4. Identification of Urinary Metabolic Profile of Individuals Occupationally Exposed to Pesticides

**Figure 5:**
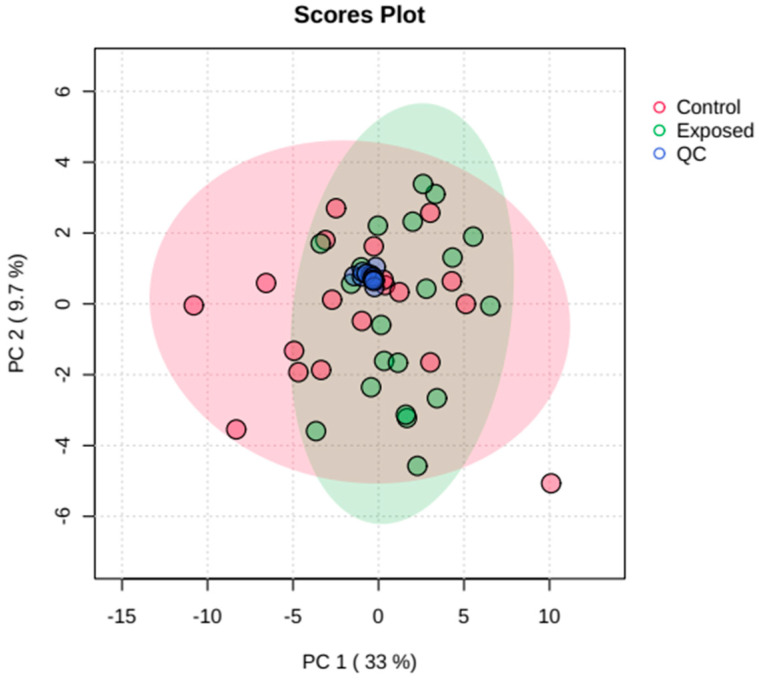
PCA model of urine samples from workers occupationally and non-occupationally exposed to pesticides as well as quality control samples (QCs).

Urine samples were prepared according to the sample preparation methodology previously described and were analyzed under the same analytical conditions, randomly, with QC samples interspersed every 5 urine samples. After the processing step (deconvolution, grouping and alignment), the data were normalized by urinary creatinine, a recommended procedure to correct the effect of variable dilutions in specific samples [59]. In sequence, they were subjected to multivariate analysis techniques. Initially, principal component analysis (PCA) was applied to all samples, including the QCs, to assess a possible separation between groups of individuals occupationally and non-occupationally exposed to pesticides. The developed model is represented in Figure 5 and shows a weak separation between the groups, but a rigorous grouping of the QCs revealed analytical stability and good quality of the acquired data.

**Figure 6:**
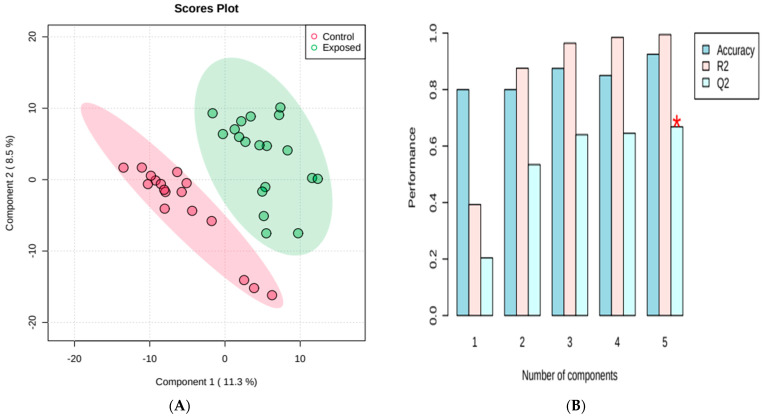
(**A**) PLS-DA model developed for urine samples from volunteers in this study, analyzed by LC-MS. (**B**) Validation of the PLS-DA model by cross-validation, using different number of components, with quality parameters R^2^ = 0.99 (variation explained by the model) and Q^2^ = 0.68 (model predictability). The red star indicates the best classifier.

Subsequently, data were analyzed using the supervised multivariate PLS-DA method. Figure 6 shows that there was a better separation between the groups.

The constructed PLS-DA model presented a good performance, with variation explained by the model (R^2^) of 0.99 and predictive capacity (Q^2^) of 0.68, for five principal components. As previously mentioned, R^2^ > 0.7 and Q^2^ > 0.4 are acceptable for biological experiments [27].

Then, the OPLS-DA model was applied, and the result is shown in Figure S3. Using this model, the constructed graphs provide better visualization of the separation between samples, but in this study, few discriminating metabolites with significant values were found (correlation and covariance > 0.8). Therefore, we decided to use the VIP values obtained in the PLS-DA analysis.

The criteria used for analysis of urine samples by LC-MS were the same as applied to plasma samples (VIP > 1.5, FDR < 0,01, *p*-value < 0.05 and fold change >2.0).

**Figure 7:**
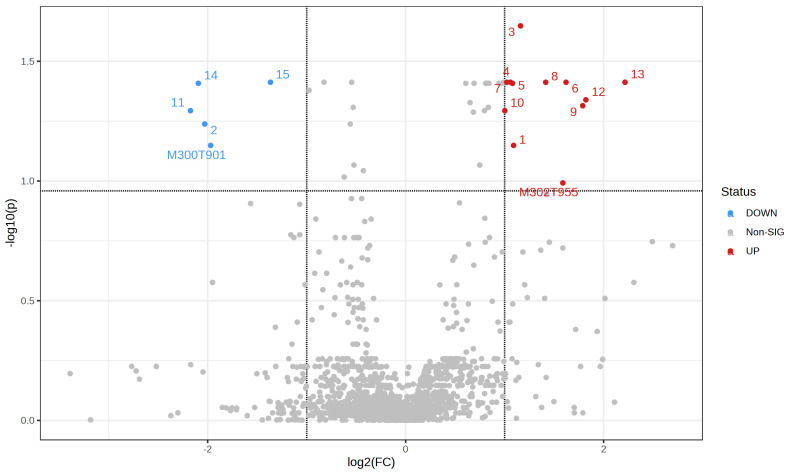
Volcano plot applied to data from urine samples of individuals occupationally exposed and not exposed to pesticides. Note: each point on the plot represents a molecular feature. The red dots indicate an increase in these and the blue dots, a decrease in them. The gray dots represent a lack of distinction between the groups. The dots identified by numbers correspond to the compounds listed in Table 3.

Based on the analysis carried out by volcano plot, 05 molecular features were found with lower results and 12 with higher results when comparing the control group in relation to the exposed one, as shown in Figure 7.

The identity of these metabolites was then searched on HMDB^®^, Metlin^®^ and Lipidmaps^®^ databases, considering a maximum mass error of 5 ppm. Two molecular features were not identified by the databases. Table 3 shows the data compiled from this evaluation and the possible identification of substances.

In order to verify the performance of selected metabolites as possible biomarkers, the ROC curve analysis was applied, and the results of some compounds are shown in Figure S4.

Based on these results, only the compounds glycosyl-2-{6-(2-cyanophenoxy) pyrimidine-4-yloxy} benzoate and carbendazim showed AUC > 0.85. The first compound (AUC = 0.88) is a metabolite of azoxystrobin, a fungicide also described in the list of substances used by our exposed group. Carbendazim, despite not being on the list of applied products, showed high levels in workers.

Azoxystrobin and other substances of the strobilurin class inhibit mitochondrial respiration by blocking electron transport. They bind to the quinol binding site of the cytochrome b-c1 complex, where ubiquinone (Coenzyme Q10) would normally bind when transporting electrons to this protein. Several animal studies have reported that this compound has the potential for developmental toxicity and neurotoxicity and may be associated with autism, brain aging, neurodegeneration, apoptosis and oxidative stress [60,61].

Mesnage et al. [62] investigated metabolic disturbances in rats by UPLC-MS caused by a mixture of six pesticides, including azoxystrobin, to obtain information on toxicity mechanisms that could act as early biochemical markers of chronic harmful effects. The authors found increased levels of some amino acids in the group exposed to the mixture of xenobiotics, in addition to decreased levels of glycerolipids, results that are in line with our findings. They concluded that there was oxidative stress resulting from exposure to the mixture of pesticides. In addition, they claimed that transcriptomic and metabolomic approaches to risk assessment procedures can result in greater sensitivity, accuracy and predictability of results, with positive implications for public health.

Bauer et al. [63] performed a UPLC-QTOF-MS screening to identify and characterize metabolites of thiacloprid, azoxystrobin and difenoconazole pesticides in plant crops and food. They addressed the degradation pathways of these xenobiotics during a kinetic study, in addition to the degradation of the original compounds. Among the results obtained, one of the metabolites detected was glycosyl 2-{6-(2-cyanophenoxy) pyrimidine-4-yloxy} benzoate. The authors mentioned that the metabolites found in the study are generally not detected in routine analyses, as these normally include only predefined metabolites and active compounds. They declared that the developed method provided new and important information about the presence and distribution of compounds related to the metabolism of xenobiotics.

Another compound that showed discriminant performance was carbendazim (AUC = 0.86), a broad-spectrum fungicide which has systemic activity of inhibiting the formation of mitotic microtubules during mitosis, affecting the growth and division of spores. According to the literature, this compound is known to manifest embryotoxicity, germ cell apoptosis, teratogenesis and infertility in different species of mammals. It is considered a mutagenic, carcinogenic and toxic agent for development and reproduction [64,65].

Chen et al. [66] used UPLC-MS metabolomic analysis to understand the effects of carbendazim on bee brain metabolism. The authors found, among the positively regulated compounds, carbendazim as one of the most abundant. In addition, they detected glycerolipids with decreased levels in bees exposed to the xenobiotic. According to the authors, the affected metabolic pathways included changes in amino acid metabolism, lipid metabolism, energy metabolism and ubiquinone biosynthesis. These results are in line with those found in our study, where carbendazim was detected at high levels and glycerophospholipids at reduced concentrations in samples from the exposed group, in addition to showing alterations in similar metabolic pathways.

Yang et al. [67] studied the risks of exposure to chlorothalonil, carbendazim, prochloraz and their mixtures in embryonic and larval zebrafish based on metabolomic analysis by LC-MS. The authors detected 26 altered metabolites, which were mainly associated with glycolysis pathways, amino acids and lipid metabolism. According to the authors, amino acids and glucose play important roles in the embryonic development of zebrafish, so the metabolomic analysis provided some important information for understanding the presumed mechanism of the three fungicides studied in aquatic organisms.

Despite the unsatisfactory performance of the other metabolites as possible discriminating compounds, evidenced by the ROC curve analysis, they are important to understand the metabolic disturbances induced by exposure to pesticides.

### 3.5. Analysis of Metabolic Pathways Affected by Exposure to Pesticides Based on Urine Metabolomics Results by UPLC-QTOF-MS

To understand the metabolic pathways involved in the biological response generated by exposure to pesticides, the variables identified and responsible for discriminating between groups were selected and then subjected to metabolic pathway analysis using the MetaboAnalyst^®^ software.

**Figure 8:**
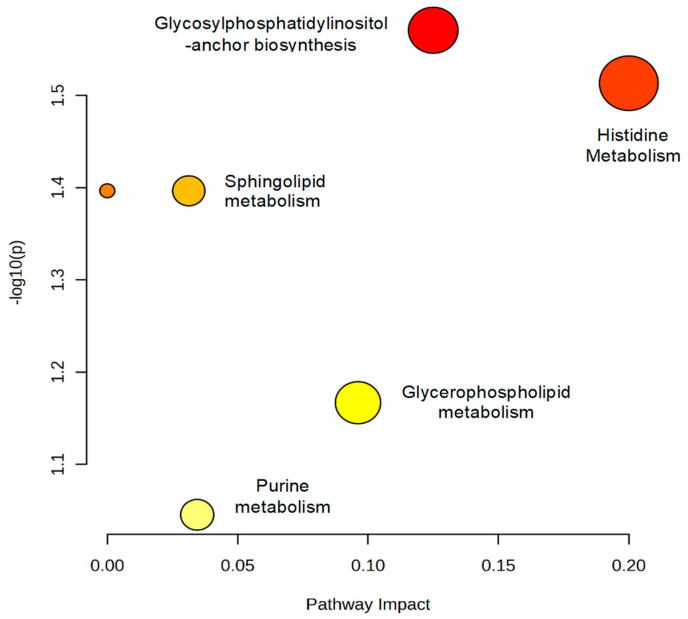
Representation of the metabolic pathways involved in the biological response associated with exposure to pesticides. The colors, varying from yellow to red, represent metabolites with different levels of significance.

All corresponding pathways according to the impact values and the *p*-values from the pathway enrichment analysis are plotted in Figure 8. Larger red circles are considered the most influenced pathways.

As shown in Figure 8, alterations were found in six metabolic pathways, and the main changes were in glycosylphosphatidylinositol-anchor biosynthesis, histidine metabolism, sphingolipid metabolism, glycerophospholipid metabolism and purine metabolism.

#### 3.5.1. Glycosylphosphatidylinositol-Anchor Biosynthesis

Glycosylphosphatidylinositols (GPI) are glycophospholipid structures that act as membrane anchors for many cell surface proteins and are essential for cell viability. The central structure is formed by a lipid group, an inositol group, glucosamine and phosphoethanolamine. The entire structure is anchored to the cell surface by the insertion of phosphatidylinositol fatty acid chains into the membrane bilayer. GPI-anchored proteins and glycoproteins play an essential role in many biological processes, such as cell recognition, activation and interaction, cell surface enzymatic reaction, embryogenesis, fertilization and bacterial and viral infection [68,69,70].

According to the literature, glycosylphosphatidylinositols can be cleaved by specific phospholipases and the protein can be released. When GPI biosynthesis is defective, these enzymes may not function properly. Studies on the regulation of phospholipid synthesis have focused on the regulation of phosphatidylinositol and phosphatidylcholine synthesis by regulating structural genes in response to the lipid precursors inositol and choline. Variants in specific genes are responsible for defects in glycosylphosphatidylinositol biosynthesis and are associated with broad clinical features, including developmental delay, intellectual disability, seizures and various congenital anomalies [70,71]. In the present study, carried out on the urine of individuals occupationally exposed and not exposed to pesticides, glycerolipids, in general, were reduced in the exposed group in relation to the control group.

Szewczyk et al. [20] used metabolomic studies to understand the influence of xenobiotics on a fungal organism through exposure to the herbicide atrazine. They observed that there was an increase in the perturbation of the membrane, causing greater membrane fluidity, probably due to a decrease in phosphatidylethanolamines. In addition, the authors mentioned that phospholipids containing inositol served as precursors for the synthesis of phosphoinositides and inositol polyphosphates and were involved in the anchoring process of plasmatic membrane proteins. They believe that a reduced level of inositol indicates lower cell viability. With the analysis of all the results obtained, they revealed that the presence of atrazine in the fungal culture induced oxidative stress, disturbances of amino acid and lipid metabolism and caused an increase in membrane fluidity.

Bernat et al. [72] verified the response of a fungal strain to the herbicide 2,4-dichlorophenoxyacetic acid, regarding the metabolome, membrane fluidity and oxidative stress. The authors observed that in the presence of the toxic compound, there were increases of up to 3 times in the permeability of the membrane when compared to the control group, indicating a significant influence of the compound in this region, and explained that the membrane can be a potential target for the action of this xenobiotic due to its lipophilicity. With this study, the authors demonstrated that the herbicide altered the general concentrations of amino acids and the profiles of fatty acids and lipids, in addition to disturbing the homeostasis of the fungal cell membrane.

The metabolic pathways of sphingolipids and glycerolipids, classes already contextualized in Section 3.3.1, were identified as being negatively altered in the urine samples of individuals occupationally exposed to pesticides.

#### 3.5.2. Histidine Metabolism

Histidine is an amino acid that has several roles in cellular function. It is involved in the biosynthesis of purinergic bases, plays a structural and catalytic role in many enzymes and has important anti-inflammatory, antioxidant and anti-secretory functions in the body. Histidine, by the action of the enzyme histidine decarboxylase, is converted into histamine, a potent mediator of numerous physiological reactions. Histamine is considered a neurotransmitter with different functions in various disorders of the central nervous system, including insomnia, Parkinson’s disease, schizophrenia, Alzheimer’s disease and cerebral ischemia [73,74].

Liu et al. [75] studied metabolic disorders in mice that were exposed to residues of common pesticides in the diet (chlorfenapyr and acetamiprid), using global metabolomics. They observed that the exposed group presented, among other changes, decreased levels of histamine, resulting in the accumulation of histidine in the body. With the analysis of all results, they concluded that exposure to xenobiotics, even at low concentrations, causes significant changes in the metabolic profile of individuals and that pesticide residues in the diet cause underestimated influences on body health.

In the study carried out by Yan et al. [76], the metabolic profile of individuals exposed to pesticides was evaluated by LC-MS. They found disturbances in metabolic pathways related to oxidative stress, inflammation, lipid and fatty acid metabolism, mitochondrial energy metabolism and neurotransmitter precursors. Among the results described, changes in histidine metabolism were observed, and the authors associated these changes with inflammation and oxidative stress. They claimed that xenobiotics may exert influences on inflammation-related pathways as well.

#### 3.5.3. Purine Metabolism

Among the metabolic pathways influenced by exposure to pesticides, the metabolism of purines was also pointed out, where lower levels of phosphoribosylamine were found in individuals in the exposed group.

Purines are nitrogenous bases that, in addition to being used in the synthesis of DNA and RNA, are important components of several biomolecules, such as ATP, GTP, cAMP, NADH and Coenzyme A. According to the literature, purine biosynthesis requires ten enzymatic transformations to generate inosine monophosphate, and the first step involves phosphoribosylamine, formed from the conversion of 5-phosphoribosyl pyrophosphate by amido-phosphoribosyl-transferase. Phosphorribosylamine is a carbohydrate derivative belonging to the class of organic compounds known as pentose phosphates. The dysregulation of purine biosynthesis has been associated with cancer, gout, neuropathologies and immunological disorders [77,78]. According to McCune et al. [79], the inosine monophosphate generated in the synthesis of purines contributes to the production of several intermediates, such as AMP, GMP, adenosine and inosine. The decrease in its production leads to a negative feedback inhibition of 5-phosphoribosyl pyrophosphate and prevents the activation, for example, of T-cells, reducing the action of the immune system.

Zhang et al. [80] developed an untargeted metabolomic method to investigate the mechanism of enantioselective toxicity of the insecticide dinotefuran in bees. They observed that the most disturbed pathway was the synthesis/metabolism of purines, associated with energy supply, and deepened the study of the effects of this toxicant on purine-related metabolites. They found 17 up-regulated and 11 down-regulated compounds in the dinotefuran-treated group. Among the metabolites with reduced levels were guanosine monophosphate and inosine monophosphate, indicating alterations in the first stages of purine synthesis. Analyzing all the results obtained in the study, it was indicated that the greatest toxicity of the pesticide was related to the disturbance of the metabolic pathway of purines and its inhibitory role in energy metabolism, concluding that this xenobiotic can endanger the existence of bees by interrupting energetic metabolism.

Kislitskaya et al. [81] analyzed disorders of antioxidant enzymes and purine metabolism in the ejaculate of men exposed to pesticides and air pollutants. The authors claimed that this exposure disturbs the balance of lipid peroxidation and antioxidant activity, activating the formation of free radicals in male germ cells, which leads to increased levels of oxidative stress and decreased purine metabolism. They concluded that these changes may interfere with morphological differentiation and sperm movement.

In general, analyzing all the results of the pathway analysis of this study, we can infer that exposure to pesticides produces toxicity through multiple mechanisms, mainly through oxidative stress, inflammatory reactions and mitochondrial dysfunction.

According to the information provided by the participants of the occupationally exposed group through a questionnaire, important alterations in organic systems were observed. Disturbances have been observed mainly in the central nervous system and peripheral nervous system, where 55% of participants reported symptoms such as headache, muscle weakness and tremors, dizziness and tingling. Alterations in the digestive system were also pointed out, with 50% of workers reporting nausea, heartburn/burning, vomiting, abdominal cramps and diarrhea. Arrhythmia, hypertension, difficulty breathing, nasal irritation and fatigue were identified in 20% of occupationally exposed individuals. It can be seen that the affected pathways identified by metabolomics are directly related to these effects reported by rural workers.

These findings corroborate the toxic effects arising from exposure to pesticides, according to results found by a systematic review carried out by Lopes and Albuquerque [82], where data from 116 studies were gathered that demonstrated the negative impact of exposure to these xenobiotics on human health and the environment. Among the most common symptoms reported by participants were headaches, nausea and stomach pain, dysuria, gastritis, abdominal cramps, respiratory diseases, anxiety, myalgia, irritability and depression.

In this study, we verified the metabolic profiles in plasma and urine samples of individuals occupationally exposed and not exposed to pesticides. It is important to emphasize that compared to plasma, the metabolic profile of urine represents the result of glomerular plasma filtration, reabsorption and tubular excretion; therefore, metabolite concentrations may be different. It should also be noted that this study has limitations, including the small sample size (*n* = 40), which may have been a source of random variation in the results of the 2 groups analyzed. Furthermore, the identification of the metabolites was putative: Level 2 as defined by the Metabolomics Standards Initiative (MSI).

## 4. Conclusions

The metabolomic study using the UPLC-Q-TOF-MS technique revealed metabolic disturbances in workers exposed occupationally to pesticides. The disturbances identified involved several metabolic pathways, with emphasis on the metabolism of lipids and amino acids. The multivariate analyses carried out using pattern recognition methods led to the identification of important metabolites for discrimination between the group exposed and not exposed occupationally to pesticides, 21 of which were plasmatic and 15 of which were urinary metabolites. With the analysis of the area under the curve (AUC), calculated using the ROC curve, the compounds with the greatest potential for biomarkers were revealed. Thus, it can be inferred that the metabolomic analysis contributed to the indication of possible candidates for biomarkers that may be capable of predicting damage to workers’ health at an early stage. However, for clinical use, the possible biomarkers need to be validated, using a larger number of individuals per group and following stricter protocols.

So far, there are no published works on the study of metabolites identified by the global metabolomics approach in plasma and urine of human beings exposed to pesticides. For this reason, the results of this study may be of great relevance for understanding the mechanisms of the toxic action of pesticides and for guiding studies on biomarkers of early effects, which can be used later in programs to monitor the health of workers.

## Figures and Tables

**Table 1:** Comparison of variables between exposed and control groups.

Variables	Exposed (*n* = 20)	Control (*n* = 20)	*p*-Value
Age group ^2^	*n* (%)	*n* (%)	0.118
20 a 30	2 (10.0)	8 (40.0)	
31 a 40	6 (30.0)	3 (15.0)	
41 a 55	9 (45.0)	5 (25.0)	
>55	3 (15.0)	4 (20.0)	
Scholarity (Years) ^1^	5 (4–8)	16 (12–20)	0.223
Occupation ^2^			<0.001 *
Administrative	0 (0)	15 (75)	
Family agriculture	14 (70)	0 (0)	
Applicator	6 (30)	0 (0)	
Student	0 (0)	3 (15)	
Other	0 (0)	2 (10)	

Note: ^1^ Mann–Whitney test; ^2^ Fisher’s exact test; * *p*-value < 0.05.

**Table 2:** Discriminating compounds identified in the metabolomic analysis using the UPLC-MS technique, in the plasma of individuals exposed and non-exposed occupationally to pesticides.

Identification	Compounds	m/z	^a^ VIP Score	^b^ Fold Change	^c^ *p*-Value	Chemical Classification
1	1-[2-chloro-4-(4-chlorophenoxy)phenyl]-2-(1,2,4-triazol-1-yl)ethanol	349.038	3.50	−3.8528	3.6 × 10^−24^	Triazole
2	13-bromo(...)hydroxy-tridecatrienoic acid	389.076	3.39	−2.3291	2.5 × 10^−14^	Prenol Lipids
3	PI(20:5(5Z,8Z,11Z,14Z,17Z)/0:0)	618.280	3.36	−3.620	1.9 × 10^−12^	Glycerophosphoinositol
4	Diphosphatidylglycerol	709.794	3.31	−4.0838	3.6 × 10^−12^	Phosphatidylglycerol
5	Cer(d16:1/LTE4)	692.479	3.17	−3.6229	1.2 × 10^−11^	Ceramides
6	Glutamate	147.053	2.57	−1.4101	1.2 × 10^−5^	Amino acids, peptides and analogues
7	PG(18:3(9Z,12Z,15Z)/22:4(7Z,10Z,13Z,16Z))	821.071	2.50	1.4805	1.5 × 10^−5^	Glycerophospholipids
8	Paraxantine	180.064	2.33	1.4462	3.6 × 10^−3^	Purine and derivatives
9	5-aminophthalazine-1,4-diol	194.083	2.31	2.1139	6.3 × 10^−3^	Benzodiazine
10	LysoPA(0:0/18:1(9Z))	436.258	2.15	1.1522	4.8 × 10^−4^	Glycerophospholipids
11	Sphingomyelin	730.598	2.01	2.14	4.3 × 10^−3^	Sphingolipids
12	1,22-Docosanedioic acid	370.308	2.00	2.4958	1.3 × 10^−2^	Fatty acids
13	3-(Methylthio)propanoyl-CoA	869.689	1.95	1.003	1.8 × 10^−2^	Glycerophospholipids
14	5-O-b-D- glucopyranoside	903.255	1.94	1.1131	1.6 × 10^−2^	Carbohydrates
15	13,14-Dihidro PGE1	356.256	1.93	−2.138	7.8 × 10^−3^	Lipids
16	Val Gly Asp	289.127	1.90	1.1063	1.6 × 10^−2^	Amino acids, peptides and analogues
17	Phosphatidylethanolamine (18:3)	475.269	1.79	−2.402	3.4 × 10^−2^	Phosphatidylethanolamine
18	Phosphatidylcholine (20:0/14:0)	762.092	1.78	−1.1386	2.3 × 10^−2^	Phosphatidylcholine
19	Norepinephrine sulfate	249.030	1.77	1.13	3.3 × 10^−2^	Arylsulfate
20	Phosphatidylcholine (14:1)	465.285	1.75	−2.6305	2.5 × 10^−2^	Phosphatidylcholine
21	L-tirosine	180.073	1.64	1.1367	3.4 × 10^−2^	Amino acids, peptides and analogues

Note: ^a^ VIP score was obtained from the OPLS-DA model; ^b^ negative fold change values correspond to increased compounds in the exposed group, while positive values correspond to decreased compounds in this same group; ^c^ *p*-values were calculated from the nonparametric Mann–Whitney test between groups exposed and non-exposed occupationally to pesticides.

**Table 3:** Discriminating compounds identified by UPLC-MS in the metabolomic analysis of urine samples from workers occupationally exposed and not exposed to pesticides.

Identification	Compounds	m/z	^a^ VIP Score	^b^ Fold Change	^c^ *p*-Value	Chemical Classification
1	Phosphoribosylamine	229.035	4.4742	2.8165	3.0 × 10^−2^	Pentose phosphate
2	Carbendazim	191,187	3.2886	−1.4924	6.0 × 10^−3^	Benzimidazole
3	Triacylglycerol	852.757	3.2706	1.1967	2.9 × 10^−4^	Glycerolipids
4	1-Palmityl-2-palmitoleoyl-glycero-3-phosphocholine	717.567	3.2557	1.1233	3.8 × 10^−3^	Glycerolipids
5	PE(22:6(4Z,7Z,10Z,13Z,16Z,19Z)/18:4(6Z,9Z,12Z,15Z))	783.483	3.2477	1.1553	3.9 × 10^−3^	Glycerolipids
6	N-Acetylgalactosamine	221.208	3.2183	1.637	4.5 × 10^−3^	Carbohydrates
7	TG(10:0/10:0/10:0)	919.767	3.0001	1.0771	1.0 × 10^−3^	Glycerolipids
8	SM(d18:1/12:0)	646.504	2.9576	1.0084	2.0 × 10^−3^	Sphingolipids
9	UDP-D-galacturonic acid	581.034	2.9366	1.0137	2.3 × 10^−3^	Pyrimidine nucleotide
10	TG(15:0/18:0/O-18:0)	834.804	29.296	1.0899	2.0 × 10^−2^	Glycerolipids
11	Glycosyl 2-{6-(2-cyanophenoxy)pyrimidine-4-yloxy}benzoate	495.127	2.9051	−1.291	2.0 × 10^−2^	Benzenoid
12	Diphosphoinositol tetraphosphate	819.794	2.797	1.0012	4.5 × 10^−3^	Inositol phosphate
13	PE(18:3(6Z,9Z,12Z)/P-18:0)	725.535	2.7486	1.0137	1.0 × 10^−3^	Glycerolipids
14	Mycophenolic Acid Glucuronide	496.158	2.4476	−1.276	3.4 × 10^−3^	Carbohydrates
15	Histidine	155.069	2.139	−1.1325	3.1 × 10^−3^	Amino acids, peptides and analogues

Note: ^a^ VIP score was obtained from the PLS-DA model; ^b^ negative fold change values correspond to increased compounds in the exposed group, while positive values correspond to decreased metabolites in this same group; ^c^ *p*-values were calculated from nonparametric Mann–Whitney test between groups exposed and non-exposed occupationally to pesticides.

## Data Availability

The data presented in this study are available in article and supplementary material.

## Supplementary Materials

The following supporting information can be downloaded at https://www.mdpi.com/article/10.3390/metabo13050596/s1: Table S1—Gradient elution and flow rate used in chromatographic separation; Figure S1—OPLS-DA score graph for plasma samples from individuals exposed and non-exposed occupationally to pesticides; Figure S2—ROC curves of some of the discriminating plasma metabolites identified by liquid chromatography coupled with mass spectrometry, where AUC = area under the curve, with a 95% confidence interval; Figure S3—Orthogonal partial least squares discriminant analysis (OPLS-DA) for urine samples from individuals participating in the study; Figure S4—ROC curves of some discriminating urinary analytes identified by liquid chromatography coupled with mass spectrometry. Note: AUC: area under the ROC curve, with a 95% confidence interval.
