# Supplementary material for: An Exploratory Study of the Metabolite Profiling from Pesticides Exposed Workers
Source: Metabolites. 2023 Apr 27;13(5):596. doi: 10.3390/metabo13050596 (PMC10220970; doi:10.3390/metabo13050596)
Supplement: Supplementary file 1 [file metabolites-13-00596-s001.zip › metabolites-2259868-supplementary.pdf]

Supplementary Materials:

Table S1 - Gradient elution and flow rate used in chromatographic separation.

Time (min)	Rate (mL/min)	% A	% B
0	0.400	98.0	2.0
3	0.400	98.0	2.0
8	0.400	90.0	10.0
14	0.400	50.0	50.0
14.50	0.400	0.0	100
16	0.400	0.0	100
16.50	0.400	98.0	2.0
20	0.400	98.0	2.0

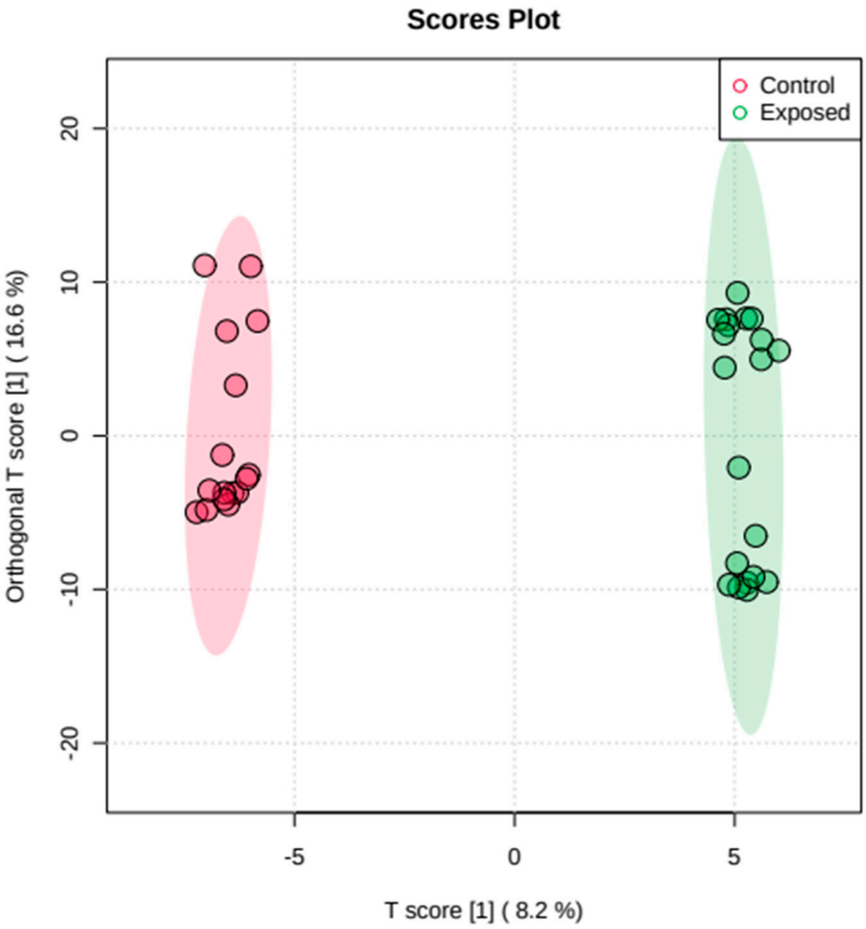

Figure S1 - OPLS-DA score graph for plasma samples from individuals exposed and non-exposed occupationally to pesticides.

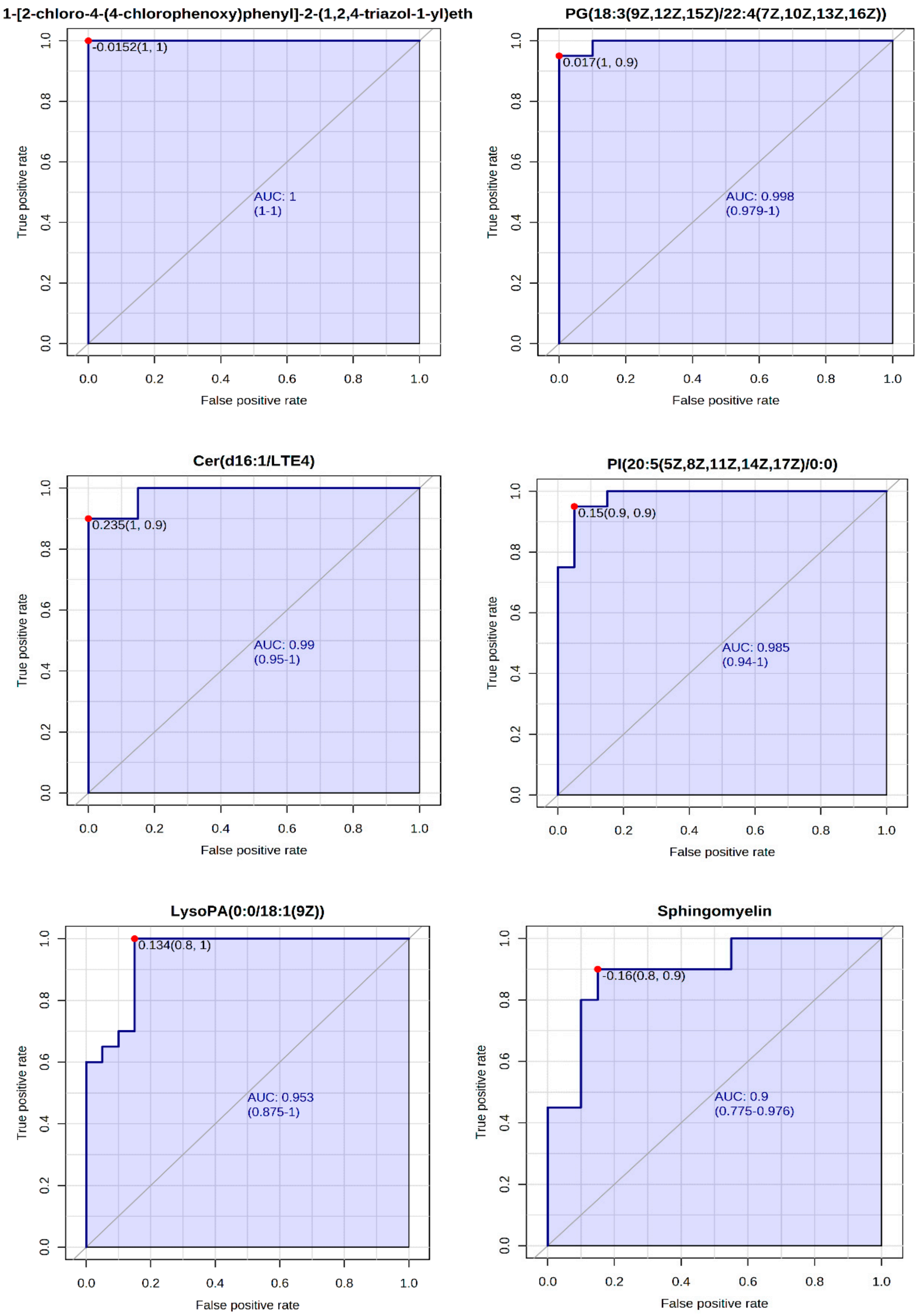

Figure S2 - ROC curves of some of the discriminating plasma compounds identified by liquid chromatography coupled to mass spectrometry, where AUC = area under the curve, with a 95% confidence interval.

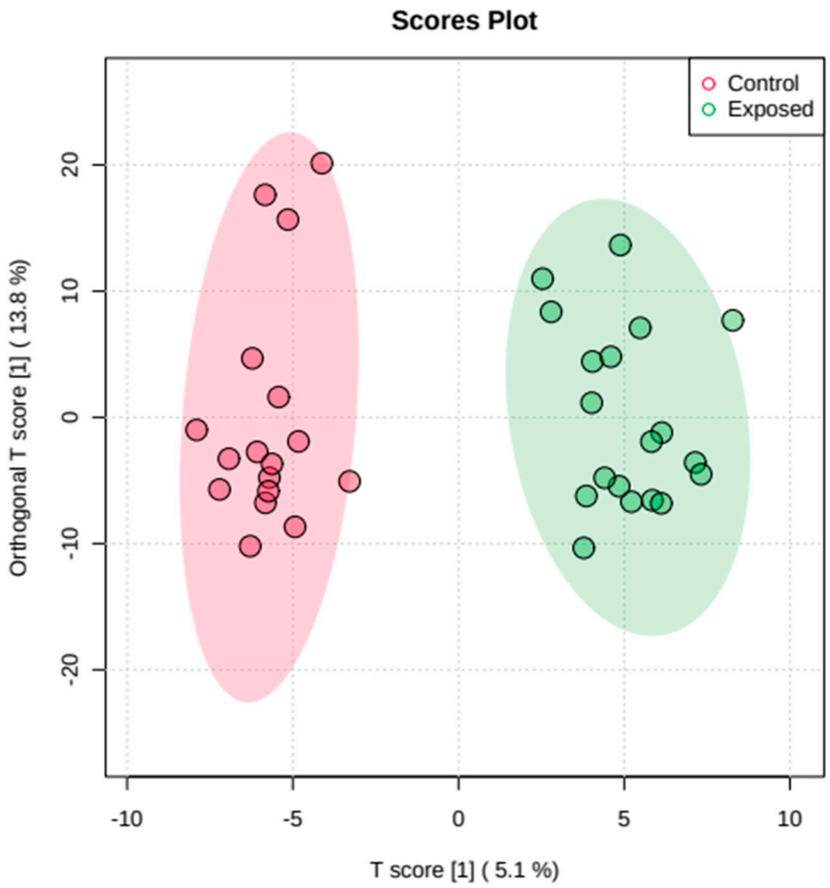

Figure S3 - Orthogonal Partial Least Squares Discriminant Analysis (OPLS-DA) for urine samples from individuals participating in the study.

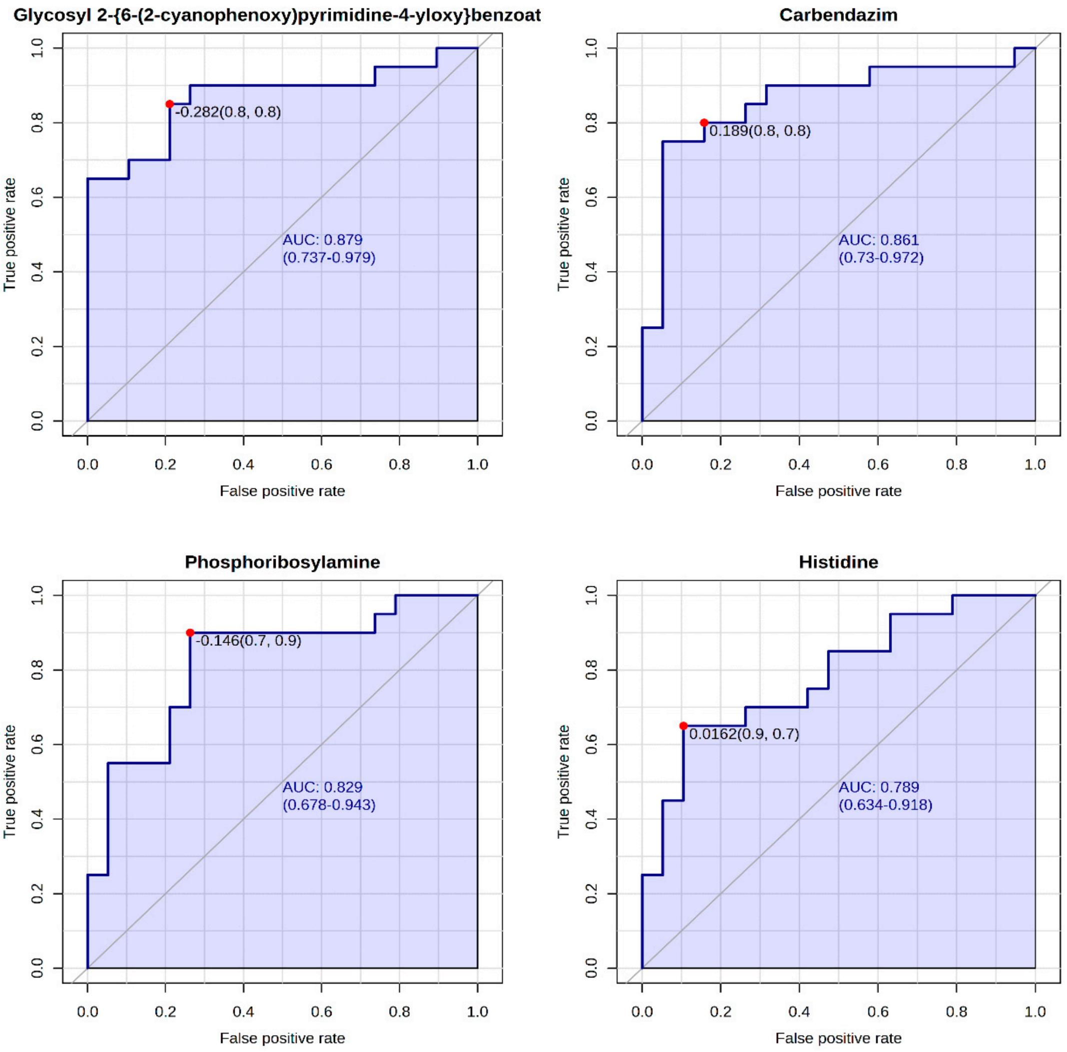

Figure S4 - ROC curves of some discriminating urinary analytes identified by liquid chromatography coupled to mass spectrometry. Note: AUC: area under the ROC curve, with a 95% confidence interval.
